# Healthy School-Aged Child With Acute Onset Altered Mental Status

**DOI:** 10.1177/00099228251356178

**Published:** 2025-07-17

**Authors:** Melissa Campbell, Michael Gracia, John Arnold, Xinyu Wang, Sydney Leibel, Sarah Gray

**Affiliations:** 1 University of California San Diego, La Jolla, CA, USA; 2 Rady Children’s Hospital San Diego, San Diego, CA, USA

**Educational Objectives**

- Group B Streptococcus (*Streptococcus agalactiae*) meningitis, though very rare, can occur during childhood.
- It is important to evaluate underlying anatomic, genetic and immunologic risk factors that may predispose a patient to infections with unusual pathogens.

## Case Report

A 7-year-old previously healthy, immunized female presented with acute onset of altered mental status and seizure-like activity. The day prior to admission, she was well and at her neurologic baseline. She transiently reported lower abdominal pain radiating to her back in the evening. Overnight she had 3 episodes of nonbloody, nonbilious emesis, without associated fever or diarrhea. Upon awakening, parents noticed that she was breathing heavily and not responsive. She had a generalized tonic-clonic seizure, lasting approximately 30 to 60 seconds. Upon emergency medical services (EMS) arrival, patient was postictal and responded only to painful stimuli. No associated rashes, rhinorrhea, congestion, diarrhea, recent illnesses, or recent travel were reported. No new medications were recently started. Exposure history was only notable for a COVID-19 exposure 1 week prior to presentation. She has history of 1 prior episode of otitis media, but otherwise she did not have any other history of significant infections in the past, including pneumonia.

On initial presentation to the emergency department, the patient was postictal with altered mental status. She was febrile, tachycardic to 154 beats per minute, tachypneic with 36 respirations per minute, normotensive, and with an oxygen saturation of 98% on room air. Her exam was notable for head tilt to the right, nuchal rigidity, positive Kernig sign, and normal upper and lower extremity reflexes. She was also noted to have 2/6 systolic murmur at the left upper sternal border.

She underwent evaluation including lab testing, blood culture, cerebral spinal fluid (CSF) analysis and culture, and head imaging. Initial white cell count was 15.0 TH/µL with 32% band neutrophils and absolute neutrophil count of 13 350 µL. C-reactive protein (CRP) was slightly elevated 4 mg/dL (normal <1 mg/dL). A respiratory viral polymerase chain reaction (PCR) panel was positive for adenovirus. CSF was noted to be cloudy with elevated nucleated cells at 5030 #/uL (96% neutrophils, 4% mononuclear cells, 0% lymphocytes), elevated erythrocytes at 339, elevated protein at 327 mg/dL, and low glucose at 37 mg/dL. Gram stain of the CSF culture demonstrated <1+ Gram-positive cocci in pairs. Computed tomography (CT) of the head and neck demonstrated a mucosal retention cyst of the left maxillary sinus with no evidence of meningeal enhancement.

She was started on broad spectrum antibiotics with intravenous (IV) ceftriaxone and vancomycin. Levetiracetam was started for seizure prophylaxis. She was admitted to the hospital for continued treatment and evaluation.

## Discussion

### Hospital Course

**Figure 1.**
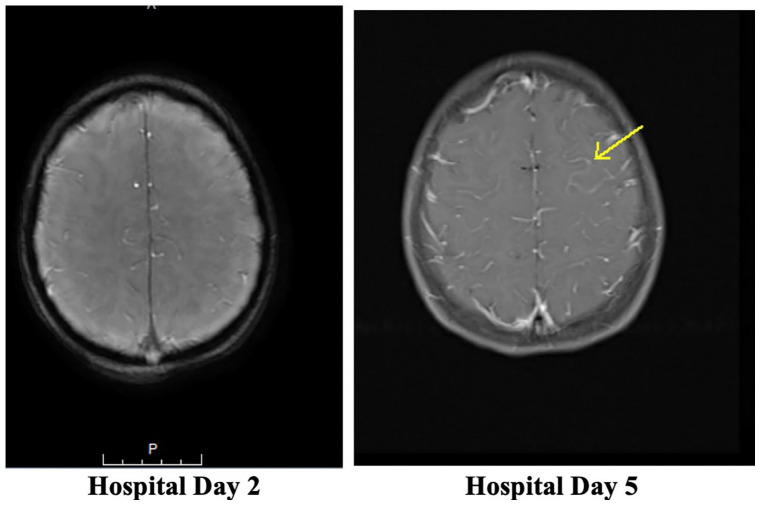
MRI Brain with contrast with diffuse FLAIR hyperintensity throughout the cerebral sulci.

**Figure 2.**
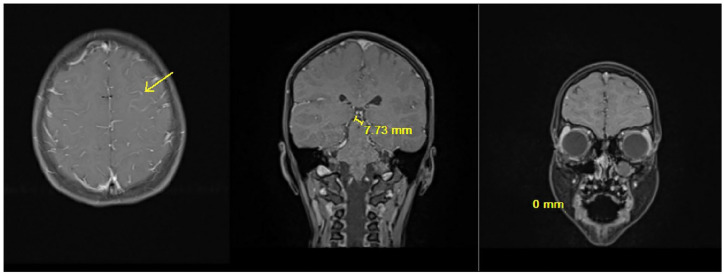
MRI Brain hospital day 5 new mild cortical reduced diffusion and edema along the left superior frontal and precentral sulci. Additional very mild edema along the left gyrus rectus. Appearance likely reflects mild ischemic injury or focal cerebritis.

The CSF meningitis/encephalitis PCR panel returned positive *for Streptococcus agalactiae* and CSF culture confirmed result, growing Group B Streptococcus (GBS). Blood culture remained negative. Infectious diseases, neurology, and allergy/immunology teams were consulted. She was transitioned to IV ampicillin and IV gentamicin. Magnetic resonance imaging (MRI) brain with and without contrast showed diffuse fluid attenuated inversion recovery (FLAIR) hyperintensity throughout cerebral sulci, otherwise unremarkable. Repeat MRI was obtained hospital day 5 and showed some signs of ischemia versus focal cerebritis compared with the initial (Figures 1 and 2). Magnetic resonance venography had no signs of thrombosis. Transthoracic echocardiogram with bubble study was normal with absence of atrial communication.

Patient gradually returned to baseline mental status, showed improved fever curve, and had normalization of serum CRP. She was found to have mild left sided hearing loss, otherwise made a full neurologic recovery.

She underwent a broad immunological evaluation given unusual pathogen in this age group. Total IgG was low at 405 mg/dL (normal 560-1319 mg/dL) and after a dose of intravenous immunoglobulin (IVIG) total IgG normalized to 665 mg/dL. IgA, IgM, and IgE were unremarkable. *Streptococcus pneumoniae* assay detected 11 of the 13 serotypes in the 13-valent conjugate vaccine, and 14 of the 23 serotypes in the 23-valent polysaccharides vaccine. *Haemophilus influenza* type B (Hib) titer was low at <0.15 mcg/mL (≥1.00 mcg/mL). Neutrophil oxidative burst assay was normal. The pediatric cellular immune panel including lymphocyte subsets, lymphocyte proliferation, antigens, and mitogens were essentially normal with minor borderline abnormalities of no clinical significance. There were some borderline findings involving certain CD-marked T cells that the immunology specialist felt represented systemic depletion during severe acute infection rather than an inherent immunodeficiency. Primary immunodeficiency disease panel found patient to be autosomal recessive for RAG1 c.1566G>T (p.Trp522Cys) deficiency.

After more than 72 hours of being afebrile on hospital day 10, she was discharged home on IV ceftriaxone to complete a total 21-day course of IV antibiotics from sterile CSF cultures. The decision for prolonged antibiotic treatment was made as a possible anatomic defect leading to central nervous system extension could not be excluded despite normal imaging. Parental consent to publish their child’s case was obtained prior to discharge.

## Discussion of Case and Literature

Group B Streptococcus meningitis outside the neonatal period or immunocompromised adults is extremely rare.^1,2^ The most common organisms to cause bacterial meningitis in school-aged children are *Streptococcus pneumoniae*, *Haemophilus influenza* type B, and *Neisseria meningitidis*.^1,2^ According to an epidemiologic study of invasive GBS disease in the United States (1999-2005) there were 233 pediatric cases of invasive GBS disease between the ages of 90 days and 14 years, with 44 meningitis cases.^3^ Of the children with GBS disease ages 1 to 14 years, 44% had an underlying condition that included neurologic disorders, immunosuppressive condition, or malignancy.^3^ A surveillance study conducted in the United States between 1998 and 2007 analyzed 587 pediatric cases of bacterial meningitis.^2^ GBS accounted for 38% of all pediatric bacterial meningitis cases in the cohort.^2^ Approximately, 52% of all pediatric bacterial meningitis cases less than 23 months of age and only 4% in children aged 2 to 17 years were caused by GBS.^2^

Neonatal rates of early onset GBS sepsis occurring in the first month of life (as a surrogate for GBS meningitis) are between 30% and 43% according to 2 sources.^4,5^ Comparatively, a study of 231 children aged 1 to 19 years (median age 7 months) identified GBS as cause of bacterial meningitis in 18.2% of cases.^6^ As the median age suggests, etiology is higher in younger children than the school-aged child described in our case report. In addition, a Canadian study of 104 all-comer children with bacterial meningitis (median age 2.2 years) found that GBS accounted for 13% of cases, demonstrating the decreased incidence as median age increases.^7^ Finally, a population study in England and Wales reviewing 7061 persons of all ages with bacterial meningitis found GBS to account for only 5% of cases.^8^ There is overall limited to no published data to the authors knowledge that specifically estimates the actual incidence of GBS in school-aged children. Our patient is one of very few reports of GBS meningitis outside of the neonatal period in a previously healthy school-aged child.

Outside of the neonatal period, risk factors for GBS meningitis include disruptions in the endothelial lining of the blood brain barrier or anatomical defects that allow GBS to spread from locations where it is normal flora (colon, female genital tract) to the CSF.^9^ Complicated bacterial infections in close proximity to the central nervous system can increase the risk of meningitis.^10^ Anatomical defects such as congenital dermal sinus tracts have also been known to increase risk of bacterial meningitis in children.^11^ Our patient had isolated mucosal retention cyst of the left maxillary sinus but without spread to the meninges.

Respiratory viral infections can lead to subsequent or concomitant bacterial infections due to disruption of respiratory tract epithelium, improved bacterial adherence and bacterial-cellular interaction.^12^ Our patient was adenovirus positive on respiratory multiplex testing. There is a low risk of developing bacterial meningitis or bacteremia from a respiratory viral illness, including adenovirus, in children.^13,14^ To our knowledge, there are no reported cases of adenovirus predisposing GBS meningitis.

Given GBS is an unusual pathogen in this age group, it is important to assess immunologic and genetic risk factors.^15-17^ Our patient was found to be a carrier for autosomal recessive RAG1 c.1566G>T (p.Trp522Cys) deficiency. Carrier status is insufficient to cause disease. Studies have reported 50% to 60% reduction in RAG complex recombination activity due to this RAG1 missense variant.^18,19^ Pathogenic homozygous or compound heterozygous for 2 missense mutations in RAG1 (p.Trp522Cys) gene have been found in persons with atypical SCID/Omenn syndrome, late-onset combined immunodeficiency disease (CID), or midline granulomatous disease.^18-21^ Although lymphocyte quantification revealed mild reductions in some T cell subsets, this was likely due to the patient’s acute illness rather than a true baseline lymphopenia.

Regarding humoral immunity, patient had initial hypogammaglobulinemia likely due to consumption from acute infection or could indicate baseline hypogammaglobulinemia. Genetic testing, normal IgA and IgM levels, and normalization of IgG argue against common variable immunodeficiency (CVID) diagnosis. With intact neutrophil function, there was no evidence for chronic granulomatous disease. Low vaccine titers may be from waning immunity from early childhood vaccinations although specific antibody deficiency should be considered and requires ongoing follow-up.

## Final Diagnosis

Healthy school-aged child with acute onset altered mental status with seizure due to GBS meningitis without evidence of an underlying significant predisposing factor. Primary immunodeficiency disease genetic panel found the patient to be autosomal recessive for RAG1 c.1566G>T (p.Trp522Cys) deficiency, but recessive mutation was felt insufficient to cause this specific presentation.

## Conclusion

Group B Streptococcus is an extremely rare cause of meningitis outside of the neonatal period, elderly, or immunocompromised. We report a case of a previously healthy immunized 7-year-old female presenting with fever, seizure, and altered mental status who was found to have GBS meningitis. The patient was treated with IV antibiotics and made a complete neurologic recovery except for residual left sided hearing loss. She underwent further evaluation to try to identify an underlying risk factor for having severe invasive disease with an unusual pathogen in her age group, but a specific causative etiology was not identified. Overall, GBS meningitis in an otherwise healthy school-aged child is extremely rare. This case highlights the importance for ongoing diligence in the evaluation of underlying risk factors for meningitis in patients found to have unexpected severe infections or unusual pathogens, given its ongoing etiological obscurity. Further investigations in cases such as this can include immunologic, anatomic, and genetic testing. More research is needed to identify factors relating to GBS disease in this age group, whether it be due to stronger virulence, undeclared genetic mutations, anatomic abnormalities, viral coinfection, or under reporting.

## Author Contributions

All authors made a substantial contribution to this project whether it was to the concept, acquisition or interpretation of the data. All authors reviewed drafts of the project and provided critical revisions and approved the final version to be published.
